# The Lipid Invasion Model: Growing Evidence for This New Explanation of Alzheimer’s Disease

**DOI:** 10.3233/JAD-221175

**Published:** 2023-07-18

**Authors:** Jonathan D’Arcy Rudge

**Affiliations:** School of Biological Sciences, University of Reading, Berks, UK

**Keywords:** Alzheimer’s disease, amyloid, apolipoproteins, blood-brain barrier, cholesterol, ethanol, lipids, lipoproteins, neurofibrillary tangles, nonesterified fatty acids, plaque

## Abstract

The Lipid Invasion Model (LIM) is a new hypothesis for Alzheimer’s disease (AD) which argues that AD is a result of external lipid invasion to the brain, following damage to the blood-brain barrier (BBB). The LIM provides a comprehensive explanation of the observed neuropathologies associated with the disease, including the lipid irregularities first described by Alois Alzheimer himself, and accounts for the wide range of risk factors now identified with AD, all of which are also associated with damage to the BBB. This article summarizes the main arguments of the LIM, and new evidence and arguments in support of it. The LIM incorporates and extends the amyloid hypothesis, the current main explanation of the disease, but argues that the greatest cause of late-onset AD is not amyloid-β (Aβ) but bad cholesterol and free fatty acids, let into the brain by a damaged BBB. It suggests that the focus on Aβ is the reason why we have made so little progress in treating the disease in the last 30 years. As well as offering new perspectives for further research into the diagnosis, prevention, and treatment of AD, based on protecting and repairing the BBB, the LIM provides potential new insights into other neurodegenerative diseases such as Parkinson’s disease and amyotrophic lateral sclerosis/motor neuron disease.

Alzheimer’s disease (AD) is one of the greatest challenges facing humanity today. It is the most common cause of dementia, accounting for around two-thirds of cases [1], with a global prevalence of around 24,000,000 people [2], mostly in Western countries, a figure that is growing as life expectancy continues to rise around the world [3].

There are currently no treatments that will prevent people getting the disease [4, 5], and current treatments can only delay disease progression by several months at best [6]. 99% of drug trials have failed [7], and many large pharmaceutical companies have abandoned research into AD therapies [8].

Why have we made so little progress in treating AD? An increasing number of researchers are arguing that it is because the current predominant explanation, the amyloid hypothesis, does not fully account for the disease [6, 9–11].

This paper argues that there is increasing evidence that the Lipid Invasion Model is a viable alternative hypothesis for AD.

## THE AMYLOID HYPOTHESIS

The amyloid hypothesis says that AD is caused by excessive levels of a protein fragment (or peptide) amyloid-β in the brain [12]. These excessive levels of Aβ cause the amyloid plaques and tau tangles, synaptic damage, inflammatory response, and brain shrinkage that characterize AD.

Certainly, in around 5% of cases of AD, genetic mutations cause increased production of Aβ, leading to the early-onset inherited form of the disease, FAD [11–13]. However, in 95% of cases, so-called late-onset AD (LOAD), the cause of excess Aβ levels is not so clear [11].

Unlike in FAD, in many LOAD cases the brain does not show increased production of Aβ [14, 15]. This has led some researchers to propose that the cause of any excessive Aβ levels, plaques, and other Aβ aggregates may be the result of abnormally low removal of Aβ from the brain, rather than of Aβ overproduction [16–21]. At the same time, other researchers argue that there would seem to be more than an adequate number of alternative mechanisms for eradicating excess cerebral Aβ from the brain [22–25].

As well as difficulties explaining the cause of excess Aβ in LOAD, there are also doubts about how critical Aβ is to LOAD progression.

Aβ is the key component of plaques, and according to the amyloid hypothesis, plaques are a key indicator of AD. However, some recent research has shown that in many cases of LOAD, the brains have low levels of plaques [26], and that often plaques are located mainly in parts of the brain that are not associated with the memory and other cognitive problems seen in AD [15, 27]. Other research has shown that substantial numbers of plaques can be found in healthy brains, without displaying any signs of LOAD [15, 28]. So, plaques do not lead to AD in all cases.

In summary, in LOAD, which accounts for 95% of AD cases, it is not at all clear that Aβ is the key to disease development or progression. Such doubts have been reinforced by the re-evaluation in 2022 of some of the original evidence for the amyloid hypothesis [29].

The failure of the amyloid hypothesis to fully account for LOAD, many researchers argue, is the reason why, 30 years after the hypothesis first emerged, there are no truly effective treatments, and why the four most commonly used drugs for treating AD have no link to the amyloid hypothesis [30–32], three of them being derived from a previous theory, the cholinergic hypothesis [33].

A number of alternative hypotheses have since emerged, which attempt to provide a better explanation for AD. One of these is the Aβ oligomer hypothesis, which argues that smaller, more soluble Aβ aggregates, rather than amyloid plaques, are the main source of AD neuropathology. However, this only overcomes some of the shortcomings of the original amyloid hypothesis [34]. Another is the tau hypothesis [34, 35], which argues for a central role for tau protein rather than Aβ in the pathogenesis of AD. Others have proposed that it is a form of lysosomal storage disorder [36] or a novel form of diabetes [37], or that AD is caused by autophagic dysregulation [38], neuroimmunomodulation [39], or by excess exposure to aluminum [40]. However, in contrast to the LIM, none of these hypotheses provides a comprehensive account of the disease pathology and risk factors.

## A NEW HYPOTHESIS: THE LIPID INVASION MODEL

The rest of this article summarizes the LIM, and how it better accounts for all aspects of AD pathology and all risk factors associated with it.

**Fig. 1:**
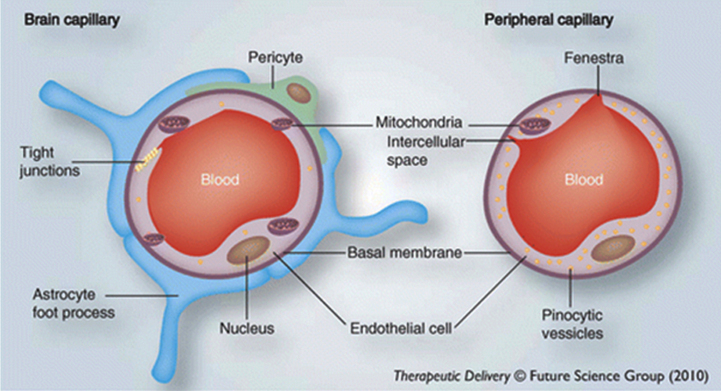
The Blood-brain barrier. The image on the left shows that brain capillaries have a protective layer and other structural arrangements that form the blood-brain barrier, whereas the image on the right shows that peripheral capillaries lack this. Used with permission of Future Science Ltd., from Mittapalli RK, Manda VK, Adkins CE, Geldenhuys WJ, Lockman PR (2010) Exploiting nutrient transporters at the blood– brain barrier to improve brain distribution of small molecules. *Ther Deliv* **1**, 775– 784 [48]; permission conveyed through Copyright Clearance Center, Inc.

The LIM argues that AD is driven by external lipids entering the brain, as a result of damage to the blood-brain barrier. The BBB is a thick protective layer (Fig. 1) around the millions of tiny blood capillaries throughout the human brain, which prevents many substances from getting into the brain tissue (or out of it) [16, 41–45]. There are occasions when it temporarily becomes more permeable to some previously-excluded substances, including solutes, cytokines and lymphocytes, due to systemic inflammation, but it restores to normal within hours or days of inflammation terminating [46, 47].

Lipids are fatty substances found throughout the body [49], and there is a different system for transporting them in the brain to elsewhere in the body. Lipids in the brain are exclusively transported by lipoproteins [50, 51], whereas lipids in the rest of the body can be transported either inside of lipoproteins or independently of them. An important role of the BBB is that it separates these two different lipid transport systems [52–55].

## LIPOPROTEINS

**Fig. 2:**
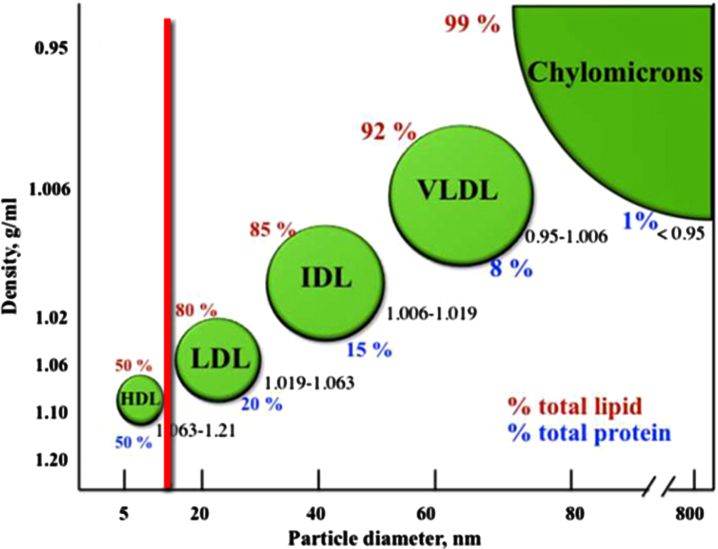
Lipoprotein classes in the bloodstream. The classification of the major types of lipoproteins is based on their densities. The density range for each class is shown, in addition to the lipid (red) and protein (blue) content. The diagram is not to scale. Image source: [56]; licensed under CC BY 3.0, with modifications by Jonathan Rudge.

Lipoproteins could be described as lipid transport containers. As can be seen in Fig. 2, lipoproteins come in different sizes, reflecting the volume of lipids they contain [51].

Lipoproteins play a key role in heart disease [57]. The smallest lipoprotein, high-density lipoprotein (HDL), is commonly referred to as ‘good cholesterol’, whereas the larger ones to the right of the red line in this figure, are commonly referred to as ‘bad cholesterol’. The cholesterol is neither good nor bad in itself; it is the excess quantity of cholesterol they contain that leads low-density lipoprotein, intermediate-density lipoprotein, and very-low-density lipoprotein to being termed ‘bad’ [58–60]. (Chylomicrons are not considered ‘bad cholesterol’ because they do not contain much cholesterol, despite their large size [61].)

Extensive studies have revealed that the brain only generates HDL-sized lipoproteins (or ‘good cholesterol’) [50, 62]. These studies include measurements of lipoprotein distributions within the brain, including in astrocytes and other glial cells, and in cerebrospinal fluid, found mostly within the fluid-filled brain ventricles, which receive brain waste) [63].

By contrast, the rest of the body also generates the much larger lipoproteins (or ‘bad cholesterol’) [51]. Normally, these are prevented from entering the brain tissue by the BBB [53, 64].

The LIM argues that when the BBB gets damaged, it allows the larger lipoproteins (the ‘bad cholesterol’) into the brain, with the result that brain cells get overloaded with the excess cholesterol within them.

This is critical because it is generally accepted that excess cholesterol uptake by neurons is associated with Aβ formation, even if the exact mechanism involved is still a matter of debate [65–74]. Cholesterol in the brain is created by astrocytes and provided to neighboring neurons, via small HDL-like lipoproteins. If these lipoproteins are artificially loaded with excess cholesterol, neuronal Aβ levels rise [65]. A 2021 study [65] showed that cholesterol levels in neurons are normally kept very low, and that this inhibits Aβ accumulation (unless Aβ is being abnormally overproduced because of genetic mutations, as seen in FAD).

Collectively, the evidence strongly suggests that, if the tight astrocyte control of neuronal cholesterol is bypassed by entry of external lipoproteins (especially larger Apolipoprotein B-containing lipoproteins, i.e.,” bad cholesterol") through damaged portions of the BBB, it will result in overproduction and accumulation of Aβ.

The Aβ created by the excess cholesterol will typically lead to the creation of plaques. The excess cholesterol will also lead to tau hyperphosphorylation and tangle formation, either as a direct result of excess cholesterol or as an indirect result of excess Aβ [65, 75–79]. In addition, other evidence suggests that cholesterol of astrocyte origin may contribute to activation of microglia, probably tau-induced [79].

In other words, exposure of nerve cells to the higher levels of cholesterol found within larger lipoproteins that have entered the brain through a damaged BBB can explain the presence of amyloid plaques and tau tangles in LOAD, and may also explain the excess stimulation of microglia and neuroinflammation in LOAD [80].

## FREE FATTY ACIDS

However, the LIM argues that cholesterol is not the only external lipid driving AD— there is another type of lipid, free fatty acids (FFAs), a form of fatty acids (FAs), that may be a more important cause of the memory and other cognitive effects seen in the disease.

FAs can serve a number of functions in the body, including as a source of energy, as ligands that activate certain cell receptors, and as components of larger lipids (e.g., phospholipids) [81, 82]. FAs are also transported differently inside and outside the brain.

**Fig. 3:**
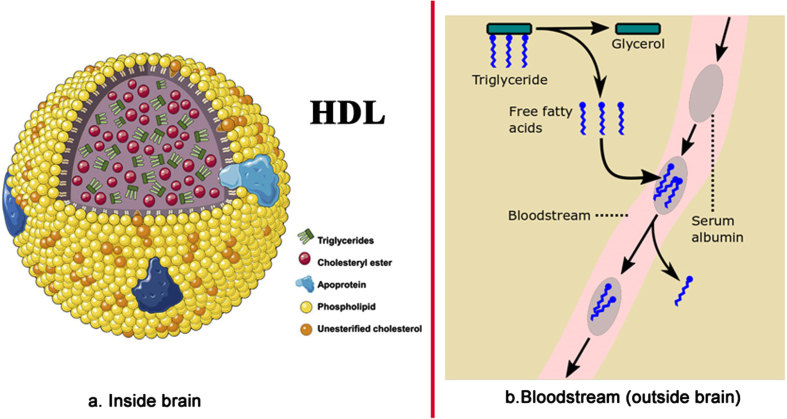
Fatty acid transport (a) inside the brain – inside HDL-sized lipoproteins, and (b) in the bloodstream outside of the brain. Image source: (a) [86], licensed under CC BY-NC-ND 4.0.

Inside the brain tissue, FAs are transported deep within lipoproteins, and similar lipid-transport particles, mostly esterified (meaning, in this case, cross-linked via a glycerol molecule) as triglycerides and diglycerides (Fig. 3a) [51]. By contrast, outside the brain tissue (primarily in the bloodstream), many FAs are transported individually, outside of lipoproteins in non-esterified form, as FFAs. These are often bound to the transporter protein serum albumin, as shown in Fig. 3b [83–85].

This difference is critical. The LIM argues that, when the BBB gets damaged, it lets in individual FFAs, which proceed to over-activate certain receptors in the brain. Most importantly these include the Toll-like receptor 4 (TLR4), involved in inflammation [87], and the extrasynaptic GABA_A_ receptor, a primary receptor for *γ*-aminobutyric acid, the major inhibitory neurotransmitter in the brain and wider central nervous system [88]. Respectively, these cause much of the neuroinflammation and anterograde amnesia (AA) seen in AD.

The effect is, in fact, similar to what happens when the brain is exposed to chronic alcohol. This is because ethanol over-activates many of the same brain receptors as FFAs, which is why it can be very difficult to distinguish AD from dementia caused by long-term alcoholism [89]. (The molecule ethanol is known to decrease neuronal excitability when administered acutely, whilst causing hyperexcitability in chronic intermittent form [90–93].) And, as in the case of ethanol-induced dementia, AD driven primarily by FFAs, rather than by cholesterol-rich external lipoproteins, will be characterized by low levels of amyloid plaques and tau tangles.

By extension, the LIM also argues that FFAs getting into the brain may be what disrupts our body clock in AD, as alcohol does [94–97], and that entry of external FFAs into the brain will drive a local ketogenic shift in brain energy production away from the normal glucose/lactate bioenergetic dominance [98, 99], as well as causing mitochondrial toxicity and oxidative stress within neurons. These are all characteristics of AD [100–104].

That is not to say that brain exposure to peripheral FFAs will be harmful in all respects. For instance, it is unclear if an FFA-driven ketogenic shift in bioenergetics would be wholly detrimental to the brain, as there is some evidence that ketone bodies may have neuroprotective properties in patients with mild to moderate AD [105–107]. And, bioenergetics aside, there is some evidence that unsaturated fatty acids, including long-chain omega-3 FFAs, may have mild neuroprotective properties in mild cognitive impairment cases and in healthy older populations [108].

However, on balance, the evidence suggests that direct large-scale exposure of brain cells to FFAs (especially saturated FFAs) will be harmful to brain cells and may help explain many AD-associated symptoms. For instance, direct exposure of the mitochondrial electron transport chain to FFAs is associated with various forms of disruption, as explained in page 139 of [109], with neuronal mitochondria likely to be at particular risk.

Exposure to high levels of certain saturated FFAs has also been shown to activate microglia, the primary immune cell of the brain, whose overactivation is known to account for much AD-associated neuroinflammation [80]. This is explained in page 139 of [109].

Finally, FFAs have been shown to induce general anesthesia in a range of animals [114–117], albeit weakly. This is explained in page 141 of [109], and suggests that exposure of the brain to FFAs will lead to AA [88], providing an alternative explanation for its early occurrence in AD. Collectively, it can be seen how external FFAs, if able to pass continually through a disrupted BBB, will cause growing damage to the brain, adding to the damage caused by excess cholesterol from larger external lipoproteins.

In conclusion, the LIM says that AD is primarily driven by the invasion of external lipids into the brain, following damage to the BBB. Entry of ‘bad cholesterol’ causes increased Aβ production, which, in turn, results in the formation of plaques and (perhaps) tangles, triggering subsequent neuroinflammation. Additionally, the increased Aβ may contribute some further BBB damage (as explained below). Entry of FFAs results also in neuroinflammation, as well as anesthesia-related inhibition of neurogenesis, anterograde amnesia and body clock disruption, neuronal mitochondrial toxicity, and changes in bioenergetics. This explains the progressive loss of neurons and other brain cells, and overall brain shrinkage.

As such, the LIM incorporates and greatly extends the amyloid hypothesis, proposing ‘bad cholesterol’ as the primary cause of the Aβ, plaques and tau tangles found in cases of LOAD, and identifying FFAs as an additional, and perhaps even more important, driver of the damage seen in the disease. Both these external lipids enter the brain tissue due to damage to the BBB. In assigning a lesser role to amyloid plaques in disease progression, the LIM explains why some cases of LOAD do not have many plaques, and why plaques do not lead to AD in all cases.

## EVIDENCE FOR THE LIM

There are three main lines of evidence that support the Lipid Invasion Model: first, the presence of lipid anomalies in AD brains; second, evidence of BBB damage in AD brains; and third the correlation between the risk factors for AD and BBB damage.

**Fig. 4:**
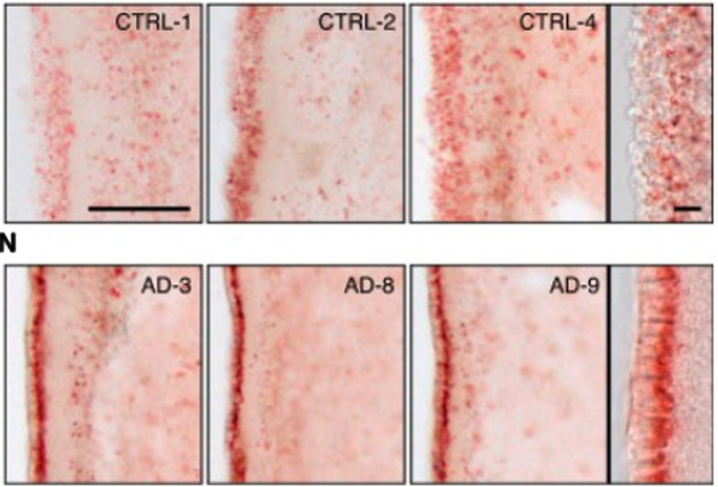
Evidence of lipid anomalies in the AD brain: (Top row) Only a few lipid droplets in ependymal cells lining the lateral ventricles of healthy patient brains; (Lower row) Many more lipid droplets in ependymal cells of AD patient brains. Panels at the right show representative higher-magnification images. Reprinted from *Cell Stem Cell*, **17**(4), Hamilton LK, Dufresne M, Joppé SE, Petryszyn S, Aumont A, Calon F, Barnabé-Heider F, Furtos A, Parent M, Chaurand P, Fernandes KJL, Aberrant lipid metabolism in the forebrain niche suppresses adult neural stem cell proliferation in an animal model of Alzheimer’s disease, 397-411, Copyright (2015), with permission from Elsevier.

First, there is abundant evidence of the presence of lipid anomalies in the brains of AD patients [77, 118–120]. This goes back to the earliest descriptions by Dr. Alois Alzheimer himself, which contain almost as many references to lipid anomalies as to plaques and tangles [119, 121]. Other early accounts reported similar anomalies [119]. An illustration of what he and his contemporaries were referring to can be seen in the two images in Fig. 4. The brain cells in the top row are normal; the brain cells with AD in the bottom row contain excessive numbers of lipid deposits, which show up in red.

Other evidence of lipid involvement in AD includes the presence of high levels of cholesterol and other lipids within amyloid plaques and in tau tangle-containing neurons in AD brains [66, 123–125]. Research in the last three years has shown that having the *APOE4* variant of *APOE* (one of the most important AD risk factors [21]) leads to faulty lipid (especially cholesterol) handling and storage in brain cells [126–128].

**Fig. 5:**
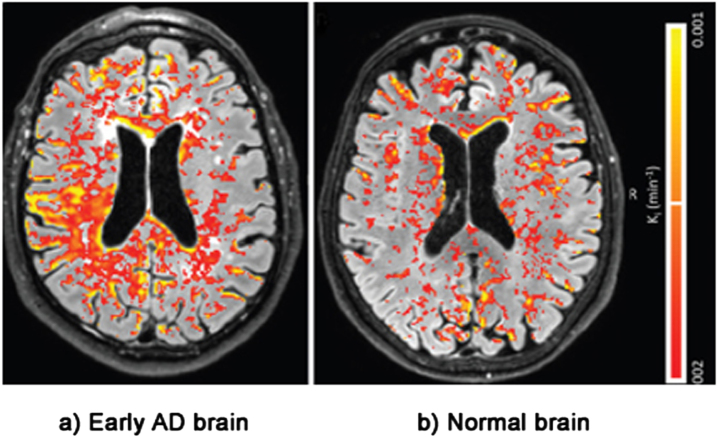
Evidence of BBB damage in the AD brain: (a) extensive leakage of gadobutrol (an MRI contrasting agent) through a damaged BBB in brains of patients with early signs of AD; (b) less extensive leakage of the agent in brains of normal patients. Used with permission of The Radiological Society of North America, from van de Haar HJ, Burgmans S, Jansen JFA, van Osch MJP, van Buchem MA, Muller M, Hofman PAM, Verhey FRJ, Backes WH, Blood-brain barrier leakage in patients with early Alzheimer disease, *Radiology* (2016) **281**, 527–535.

The second line of evidence for the LIM comes from physical evidence of BBB damage inside the brains of AD patients. This evidence is taken from postmortem brains, MRI and PET scans [16, 129–135]. Figure 5 shows an MRI scan of leakage through the BBB of biomarker gadobutrol in the brain of a patient with a mild form of cognitive impairment— early AD— compared with leakage in a normal brain. The BBB leakage in the early AD brain on the left is clearly substantially higher than the leakage in the normal brain on the right [136].

Interestingly, MRI scans of more advanced AD brains often show that the BBB has been damaged close to the location of plaques and tangles [137–139].

Other studies of AD patients [16, 130–132, 140–145] have detected the presence of proteins in the bloodstream that are normally found only in the brain tissue, and vice versa. These include the blood transport proteins Apolipoprotein B and serum albumin. Apolipoprotein B is normally found only in the larger lipoproteins (the ‘bad cholesterol’) in the external blood stream. Serum albumin is the primary transporter of FFAs, again a form of transport only found in the external bloodstream [83, 84]. Both these proteins should be stopped from entering the brain tissue by the BBB.

The third line of evidence supporting the LIM, shown in Table 1, is that all of the risk factors for AD are also risk factors for damage to the BBB. The risk factors include aging, brain trauma, high blood pressure, stress, sleep deprivation, smoking, excess alcohol, obesity, diabetes, having the *APOE4* genotype, and Aβ [146–157].

**Table 1:** Risk factors for Alzheimer’s disease are also risk factors for blood-brain barrier (BBB) damage

Risk Factor	Alzheimer’s disease	BBB Damage
Aging	✓	✓
Brain trauma	✓	✓
Hypertension	✓	✓
Stress	✓	✓
Sleep deprivation	✓	✓
Smoking	✓	✓
Excess alcohol	✓	✓
Obesity	✓	✓
Diabetes	✓	✓
*APOE4*	✓	✓
Amyloid-β	✓	✓

In LOAD (95% of AD cases), BBB damage can be caused by all the factors listed, but the primary cause is the wear and tear of aging, which is how the LIM explains why AD so disproportionately affects older people, and why the number of people affected by the disease is growing as life expectancy increases globally.

Brain trauma is also a key risk factor for BBB damage, which, the LIM argues, is why increasing numbers of contact sports players are showing signs of AD and similar dementias such as chronic traumatic encephalopathy, often at an even earlier age than FAD [158–160].

As can be seen, Aβ is also one of the risk factors for BBB damage. Aβ can damage the BBB in many ways, including: redistributing and reducing tight junction protein expression; causing the loss of pericytes, a key BBB component cell; increasing matrix metalloproteinase expression, leading to erosion of the BBB basement membrane; and promoting uncontrolled angiogenesis [41, 161–165].

In FAD AD is caused primarily by the overproduction of Aβ due to genetic mutations, as established by the amyloid hypothesis. However, the overproduction of Aβ also damages the BBB.

The fact that in FAD excess Aβ is presumably being produced from birth, and yet disease onset is typically seen only in people over 50, suggests that Aβ is only slowly damaging to the BBB, perhaps because most Aβ is locked up in plaques or other aggregates, or cleared from the brain. This may explain why attacking Aβ does not have a big impact on halting disease progression.

In summary, the LIM argues that BBB damage is the primary cause of AD overall, as shown in Table 2.

**Table 2:** Causes of AD according to the LIM

Type of AD		AD driver	Immediate biological impact	Subsequent biological impact	Final biological impact
FAD	LOAD				
–5% of cases	–95% of cases				
✓		Genes (AβPP -associated)	- Increased Aβ	- Endosomal-lysosomal disorder	- Synaptic and neuronal death
✓	✓	Invasion of ‘Bad cholesterol’ - LDL, IDL, VLDL, following BBB damage		- Amyloid plaques	- Long-term memory loss
				- Tau tangles	- Brain shrinkage and enlarged ventricles
				- Neuroinflammation- Some modest Aβ-mediated BBB disruption	- Death
✓	✓	Invasion of FFAs, following BBB damage	- Stimulation of brain receptors(e.g., TLR4, extrasynaptic GABA_A_) - Ketogenic shift in brain bioenergetics- Neuronal mitochondrial toxicity	- Neuroinflammation - Inhibition of neurogenesis - Anterograde amnesia - Body clock disruption - Oxidative stress	

AD, Alzheimer’s disease; FAD, familial AD; LOAD, late-onset AD; LDL, low-density lipoprotein; IDL, intermediate-density lipoprotein; VLDL, very-low-density lipoprotein; BBB, blood-brain barrier.

The fact that most new drug trials have been targeted at Aβ, which, according to the LIM, is not a key driver of AD progression in 95% of cases (i.e. LOAD), could be the reason why 99% of such trials have failed, and why the most successful amyloid-related drug, lecanemab (approved for medical use in January 2023), shows only modest benefits, even when administered at the earliest stages of AD [166].

## IMPLICATIONS OF THE LIM FOR AD RESEARCH

The Lipid Invasion Model has been developed from piecing together the results of hundreds of research reports on AD published over the last 40 years. There are elements that need to be tested with empirical evidence. For example, further studies are needed to confirm reports of the presence of Apolipoprotein B and excess cholesterol in close proximity to amyloid plaques and to sites of BBB disruption, and to establish the underlying mechanism linking them [65, 123, 124, 143, 145, 167]. Also, to rule out the possibility that proximity of BBB damage to elevated Aβ and plaque levels may be the result of reduced Aβ drainage, rather than of excess Aβ production. The University of Reading in the UK is researching this.

However, if the model is correct, it means that if we want to make progress in treating AD, we should reduce our research focus on Aβ and pay more attention to the BBB.

The first step is enabling identification of BBB damage using scanning and leakage markers, such as gadobutrol. This can be a good predictor of having AD, when memory problems are only just beginning to emerge, as was shown in Fig. 5.

Thereafter, research is needed to find measures to protect the BBB, and treatments to repair any damage that has already occurred. This will not be easy, given the complexity of the BBB and our limited understanding of it.

Increasing evidence suggests that encouraging lifestyle changes can help prevent BBB damage, since many of the risk factors for BBB damage and AD are also lifestyle factors (as shown in Table 1) [149, 168–171].

This is reinforced by the findings from one of the largest longitudinal studies ever within the field of dementia (29,072 adults studied over 10 years), published in January 2023 [172]. This shows that adoption of certain lifestyle factors (a healthy diet, taking regular physical exercise, not drinking alcohol and not smoking, as well as active cognitive activity and social contact) can significantly slow cognitive decline with age, both in the presence and absence of *APOE4*.

Other recent studies have suggested that certain foods and dietary supplements can protect the BBB, and even repair it [173–184]. And there has been growing interest in how a healthy, more varied diet can promote a healthy gut microbiome, which has been shown to protect and maintain the BBB [180, 185–187]. This may help to explain why the longitudinal study into cognitive decline mentioned above [172] identified diet as the most important of the lifestyle factors under study for slowing memory decline with age.

## POSSIBLE WIDER IMPLICATIONS OF THE LIM

The current version of the LIM is focused on AD. However, the model may have wider implications. The LIM suggests that external lipid invasion through a damaged BBB may well explain many cases of Parkinson’s disease and ALS/motor neuron disease. As in AD, affected brains in both diseases show lipid anomalies [188–191], and, as is reported so often in the press, participants in sports in which head trauma is a frequent event, such as boxing, football and rugby, have a higher risk of getting both these neurodegenerative diseases [158, 192–194], as well as AD and similar dementias such as chronic traumatic encephalopathy [158, 159].

## CONCLUSION

The LIM provides a new and comprehensive explanation of the neuropathologies and risk factors associated with AD, and offers new insights for future research into the disease, focusing on protecting and repairing the BBB rather than attacking Aβ.
